# Efficacy and safety of thoracoscopic-guided multiple paravertebral block for video-assisted thoracoscopic lobectomy surgery: a randomized blinded controlled study

**DOI:** 10.3389/fsurg.2023.1267477

**Published:** 2023-10-24

**Authors:** Zhixiong Li, Qingshui Lin, Liangqing Lin, Qinghua Wu, Pinhui Ke, Huan Chen, Chunlan Lin, Yaohua Yu

**Affiliations:** Department of Anesthesiology, The School of Clinical Medicine Fujian Medical University, The First Hospital of Putian, Putian, China

**Keywords:** thoracoscopic-guided paravertebral block, intraoperative analgesia, early postoperative analgesia, opioid-sparing effect, video-assisted thoracoscopic lobectomy, postoperative recovery quality

## Abstract

**Background:**

Paravertebral block (PVB) has been increasingly popular for postoperative analgesia. However, few studies estimated the efficacy and safety of multiple PVB using thoracoscope-assisted technique for intraoperative analgesia and postoperative pain management for video-assisted thoracoscopic lobectomy (VATS LOBECTOMY).

**Methods:**

A total of 120 patients scheduled to undergo VATS LOBECTOMY were randomly assigned into two groups: a placebo group and a PVB group in a ratio of 1:2. Thoracoscopic-guided multi-point PVB was carried out with 0.5% ropivacaine (PVB group) or 0.9% NaCl (placebo group) at the beginning and the end of surgery. The primary endpoint was consumption of intraoperative opioid.

**Results:**

Consumption rate of intraoperative opioids was significantly lower in the PVB group (878.14 ± 98.37 vs. 1,432.20 ± 383.53 for remifentanil; 123.83 ± 17.98 vs. 266.42 ± 41.97 for fentanyl). Postoperatively, significantly longer duration of using patient-controlled intravenous analgesia for the first time, reduced times of analgesic pump pressing, and less rescue analgetic consumption were observed in the PVB group. Visual analog scale scores at rest and during exercising were significantly lower in the PVB group at all time points within the first 48 h after surgery. The PVB group was also associated with significantly higher total QoR-40 scores and lower incidence of analgesia-related adverse events.

**Conclusions:**

Thoracoscopic-guided multiple PVB was a simple and effective technique in controlling pain both intra- and postoperatively for VATS LOBECTOMY. It was also associated with the absence of detrimental effects attributed to opioid overuse and benefits of the early resumption of activity and physical function recovery. Therefore, this regional anesthesia technique should be advocated as part of a multimodal analgesia protocol for VATS LOBECTOMY.

## Background

In recent years, the electronic video-assisted thoracoscopic surgery (VATS) has been gradually introduced to replace open thoracotomy lobectomy with several superiorities including minimally invasive technique, decreased postoperative complications and morbidity, and faster recovery (1). Nevertheless, some evidence found that moderate-to-severe acute pain is still closely associated with VATS LOBECTOMY, which impairs the ability of the patients to cough and deeply breathe resulting in respiratory complications and delayed recovery and may also develop into persistent pain syndrome following thoracotomy (2). Therefore, an effective control of peri- and postoperative pain remains a contemporary issue. Systemic reviews of analgesic technique and pain management in thoracotomy described that thoracic epidural analgesia (TEA) had been regarded as the gold standard for analgesia and satisfactory reduction of pain following thoracotomy. As a result, its use led to reduced consumption of intraoperative opioids, early extubation, better gas exchange, and decreased incidence of pneumonia (3). However, conduction of TEA and management of continuous infusion of pain medication through an epidural catheter require highly trained medical anesthetists with experience. In addition, TEA can be accompanied by neural injury, hypotension, urinary retention and postoperative nausea and vomiting (PONV) and is contraindicated in the presence of local sepsis, coagulopathy, and difficult thoracic anatomy (4). At present, multimodal analgesia (MMA) strategy using a combination of systemic analgesia and regional anesthetic block is recommended to optimize perioperative pain control and avoid the detrimental effects of opioid overuse for VATS LOBECTOMY (5). As an important representative of perioperative regional anesthesia technique, thoracic paravertebral block (TPVB) refers to a technique of injecting local anesthetic (LA) into the thoracic paravertebral space to block the intercostal nerves out of the intervertebral foramen; it blocks injury-induced activation and sensitization of both the peripheral and central nervous systems (6). In addition, TPVB is reported as a unilateral technique to preserve the pulmonary and sympathetic function in the contralateral side and cannot be precluded by contraindications to TEA (7). Results from network meta-analysis found that TPVB showed outstanding analgesic effects for postoperative analgesia for VATS as opposed to other regional anesthesia including the erector spinae block, the serratus plane block, the serratus interfascial plane block, and the intercostal nerve block (8). Recently, TPVB can be utilized under ultrasound (US) guidance with a clear pleural and lung tissue of visualization and real-time visualization of a puncture needle. Although it is safer if compared with conventional TPVB using disappearance of penetration resistance as puncture landmarks, some serious complications such as pneumothorax, inadvertent vessel puncture, or injection are considered (9), whereas PVB under thoracoscopic direct vision was technically easier and safer to perform when compared with US-guided TPVB, especially in cases when multiple or repeated implementations were required. It would be an appropriate alternative to technically produce important benefits for anesthesiologists who were not skilled in US-guided PVB. Therefore, we first conducted this randomized controlled blinded clinical trial to estimate whether the application of thoracoscopic-guided multiple-point TPVB at the beginning and at the end of the VATS LOBECTOMY could achieve an intraoperative opioid-sparing effect and an adequate postoperative analgesia after surgery.

## Methods

### Study design and patients

This randomized double-blinded placebo-controlled study was conducted with an enrollment of 120 eligible patients scheduled for VATS LOBECTOMY under general anesthesia at our department of anesthesiology between 18 April 2022 and 15 April 2023. All methods were carried out in accordance with the principles of the Declaration of Helsinki. Moreover, ethical approval was obtained from the Scientific Research Ethics Committee of Putian First Hospital affiliated to Fujian University (PTFH2022-014). All patients signed a written informed consent before their participation. The study was registered in the Chinese Clinical Trial Registry (ChiCTR2200060675).

Inclusion criteria were as follows: (1) age between 30 and 79 years and (2) classification of physical status (II–III) according to the American Society of Anesthesiologists (ASA). The exclusion criteria were the following: (1) severe cardio-cerebrovascular dysfunction, (2) a relevant anesthetic allergy, (3) chronic use of analgesics, (4) a body mass index (BMI) of ≥35 kg/m^2^, (5) contraindications for regional anesthesia, (6) psychiatric disease, and (7) refusal to participate.

### Randomization and masking

The randomization sequence was generated using a web-based system (www.randomization.com) with permuted blocks of six by two independent nurse anesthetists and concealed in sequentially opaque sealed envelopes. It would be revealed to the researchers until all analyses were completed. The anesthetic drug and placebo which could not be distinguished due to their same-colored appearance were prepared by two pharmacists not involved in the study in advance on the day of surgery.

**Figure 1:**
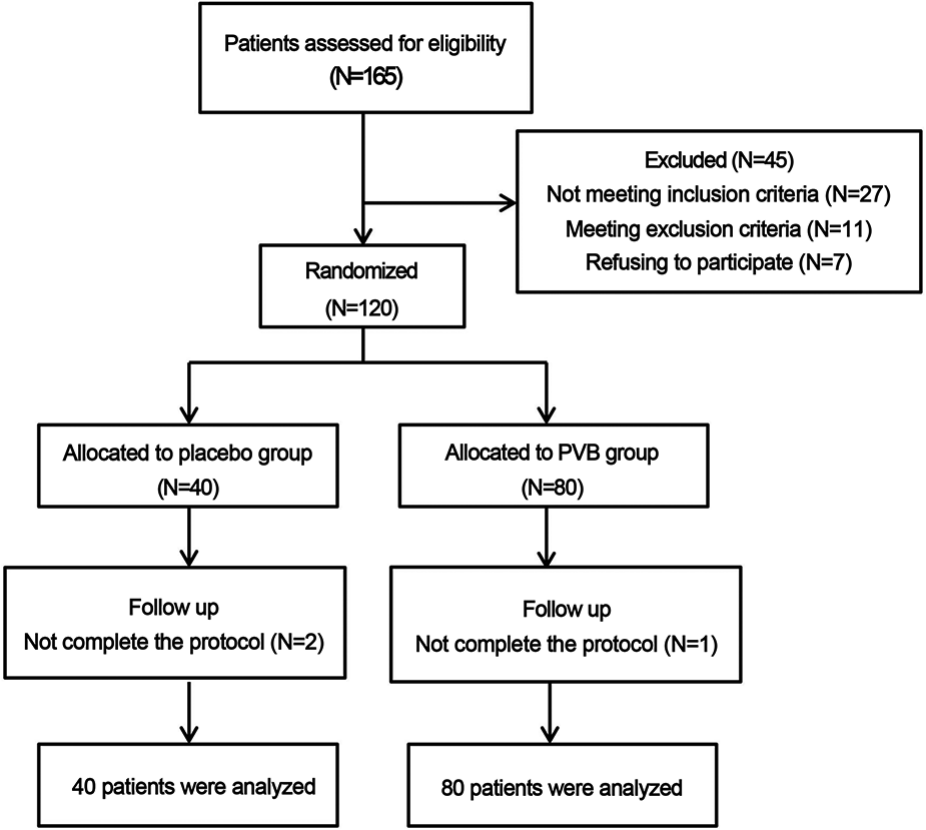
The CONSORT diagram of patient recruitment.

Patients were assigned to two groups in a ratio of 1:2: the placebo group, receiving thoracoscopic-guided placebo PVB with 10 ml of 0.9% normal saline (NS) in each space, and the PVB group, receiving thoracoscopic-guided PVB with 10 ml of 0.5% ropivacaine in each space (Figure 1). Patients, anesthesiologists, surgeons, and investigators who were responsible for the outcome assessment as well as data analysis were all masked from the group allocation. Once enrolled, the criteria for the patients to be excluded from the study were the presence of local anesthetic systemic toxicity, unpredicted conversion to open surgery, and violation of study postoperative analgesia protocol.

### Anesthesia protocol and surgery procedure

The standard anesthetic protocol of general anesthesia with endobronchial intubation was provided to all patients. Routine preoperative monitoring including electrocardiogram (ECG), non-invasive blood pressure (NIBP), oxygen saturation (SpO_2_), and bi-spectral index (BIS) were conducted upon arrival at the operating room, and both peripheral arterial and venous accesses were established. General anesthesia was induced with DEX 0.5 µg/kg, Propofol 1.5 mg/kg, sufentanil 0.5 µg/kg, and cisatracurium 0.15 mg/kg. A double-lumen endobronchial tube was placed, and an appropriate location was verified by fiber-optic bronchoscopy. Anesthesia was maintained with intravenous target-controlled infusion (TCI) of Propofol 1.5–3.0 µg/ml and remifentanil 2.5–3.5 ng/ml adjusted according to a BIS value between 40 and 60 and intermittent infusion of cisatracurium as needed. Intravenous (IV) fentanyl 1 μg/kg was administered if variations in intraoperative mean arterial pressure (MAP) and heart rate (HR) of ≥20% basal values were observed. Vasoactive agents such as atropine, ephedrine, and norepinephrine were used when necessary. One-lung ventilation and intermittent positive pressure ventilation were conducted with the maintenance of end-tidal carbon dioxide (P_ET_CO_2_) 35–40 cmH_2_O. At the end of surgery, neostigmine 1 mg and atropine 0.5 mg were administered to antagonize neuromuscular blockade, as needed.

All VATS lobectomies were performed by using a 10 mm, 30° angled HD video-thoracoscope and by using the same standardized three-port technique. A 5 cm anterior utility incision was made and protected by a plastic soft tissue retractor (Alexis Retractor, Applied Medical USA). 3-0 and 5-0 Prolene sutures were, respectively, used for reconstruction of the bronchial vascular and pulmonary arteries. A 24-Fr chest tube was placed at the seventh intercostal space through the camera incision at the end of surgery.

All patients were transferred to the post-anesthesia care unit (PACU) for the first 0.5 h postoperative observation following extubation and then transferred to an inpatient ward to receive a standard postoperative care for 48 h after completely awake.

### Paravertebral block procedure

**Figure 2:**
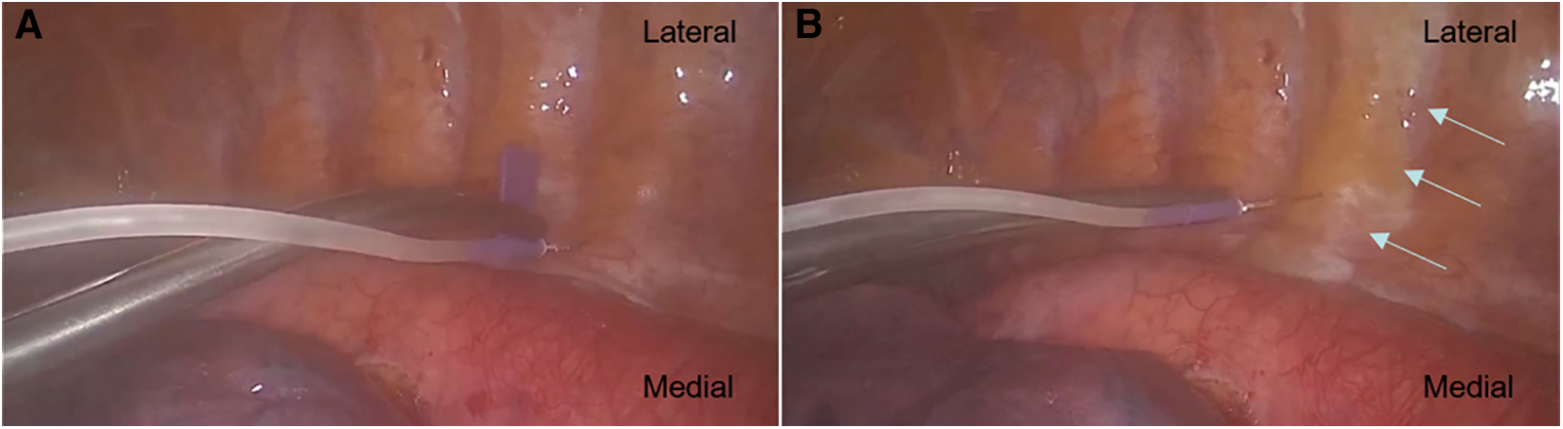
(**A**) Paravertebral block was performed at the designated space under thoracoscopic direct vision. A scalp needle was clamped by oval forceps and advanced vertically 0.5 cm beyond the parietal pleura. (**B**) The arrows showed the injected admixture spread along the targeted paravertebral space with visualization of dynamic pleura distention.

All two-space PVBs were performed by the same designated four surgeons at the T_4–5_ and T_7–8_ levels under a thoracoscopic direct vision at the beginning and at the end of the VATS LOBECTOMY, respectively (Figure 2). The puncture point was located 1 cm adjacent to the ipsilateral sympathetic chain in the pleura at approximately the middle point of the intercostal space. A scalp needle was clamped by oval forceps and advanced vertically 0.5 cm beyond the parietal pleura. After a negative aspiration of the blood and cerebrospinal fluid (CSF), a 10 ml combination of 0.5% ropivacaine was slowly injected into the targeted paravertebral space with visualization of dynamic pleura distention following the drug spread in the PVB group. Patients in the placebo group received an injection of 0.9% normal saline with the same volume for each segment. Another injection would be administrated through an alternative puncture point, if hemorrhage or hematoma occurred during the process. A lip emulsion was prepared in advance for the possible presence of LA toxicity in each patient.

### Postoperative pain management

All patients would receive a routine protocol of patient-controlled intravenous analgesia (PCIA) for 48 h after surgery with a continuous infusion of 0.03 µg/kg/h sufentanil and 5 µg/h palonosetron, and the total volume is 150 ml. In addition, the PCIA device provided a bolus delivery of 2 ml admixture with a lockout time of 10 min and a maximum limit of 40 ml within 4 h. Intravascular parecoxib (20 mg, every 6–12 h, a maximum of 80 mg/d) was permitted as rescue analgesia for all patients.

### Outcome measures and data collection

Our primary endpoint was the cumulative consumption of intraoperative opioids such as TCI remifentanil and IV fentanyl and postoperative sufentanil through PCIA in the first 48 h following VATS LOBECTOMY. The following secondary outcomes were assessed: (1) consumption of postoperative pain severity at rest and while exercising evaluated by the 11-point visual analog scale (VAS) (0 = no pain to 10 = pain as bad as you can imagine) at 2, 6, 12, 24, and 48 h upon arrival at the cardiothoracic surgical care unit (CICU) after surgery; (2) patients’ Ramsay sedation scores at 2, 6, 12, 24, and 48 h upon arrival at the CICU (score I: anxiety, anxious, and/or restless; score II: cooperative, oriented, and calm; score III: responding to instructions; score IV: brisk response to stimulus; score V: sluggish response to stimulus; score VI: no response to stimulus—an appropriate sedation level was predefined as score II–IV); (3) duration of first-time usage of PCIA pump, number of PCIA press, and rescue analgesia consumption; (4) patients’ quality of recovery within 48 h after surgery in the CICU estimated by QoR-40 questionnaire which contains five dimensions of recovery after surgery and anesthesia, namely, physiology, emotion, cognition, nociception, and activities of daily living with a five-point Likert scale for each item; and (5) adverse events of surgery and analgesic such as pleural effusion, pneumonia and pulmonary atelectasis verified by computerized tomography (CT), arrhythmia shown in ECG, hypotension, delirium, dizziness, and PONV.

### Sample size calculation

Taking the intraoperative remifentanil consumption of 1,389.32 with a standard deviation (SD) of 680.9 based on our pre-trials, we want to detect at least 30% reduction in average in the PVB group. To achieve a 90% power at the significance level of 5%, an estimated of 32 and 64 participants in the placebo group and the PVB group were needed, respectively. The current sample size of 32 and 64 participants in the placebo group and the PVB group, respectively, was determined using PASS version 16.0 software for Windows. The sample size was, respectively, 21 out of 42 and 25 out of 50, if intraoperative IV fentanyl and postoperative sufentanil through PCIA in the first 48 h was used to reject the null hypothesis. Therefore, the final sample size was 40 in the placebo group and 80 in the PVB group allowing for a possible dropout rate of 20%.

### Statistical analysis

Statistical analysis was conducted using SPSS software, version 22.0 (SPSS Inc., Chicago, IL, USA). Statistical significance was considered as *p* < 5%. Kolmogorov–Smirnov test was used to examine the normality of variables. Data were described as mean ± SD, median ± interquartile range (IQR), frequency, and percentage. General linear model (GLM) with repeated measures was employed for repeatedly measured non-normally distributed data. Mann–Whitney test was applied in comparing groups at each time point because an interaction of group by time was observed, and the Student–Newman–Keuls multiple comparison post hot test was used to differentiate within the groups. Independent *t*-test, Mann–Whitney *U* test, and *χ*^2^ test were, respectively, used to compare means, medians, and proportions. An intent-to-treat (ITT) analysis was the primary approach for all analysis. Missing values to follow-up in both groups were imputed using multiple interpolation (MI).

## Results

A total of 165 patients were identified for eligibility in the study, but 45 patients were excluded for not meeting the inclusion criteria (*n* = 27), refusing to participant (*n* = 7), or conforming to the applied exclusion criteria (*n* = 11) (Figure 1). Two patients in the placebo group and one patient in the PVB group were excluded due to unpredicted surgery conversion to open surgery and extra-intramuscular morphine injection for inadequate analgesia by frequent cough, respectively. No local anesthetic systemic toxicity occurred in any patient. Demographic characteristics of the patients were summarized in Table 1, and profiles were comparable between the two groups.

**Table 1:** Baseline characteristics of participating patients.

Variables	Control group (*n* = 40)	PVB group (*n* = 80)	*p*
Age (years)	58.51 ± 11.12	57.54 ± 9.84	0.671
Female sex, *n* (%)	21 (52.5%)	47 (58.8%)	0.561
BMI	22.9 ± 4.26	22.3 ± 2.98	0.468
ASA classification, *n* (%)			0.788
II	35 (87.5%)	68 (85.0%)	
III	5 (12.5%)	12 (15.0%)	
Smoking history, *n* (%)	8 (20.0%)	15 (18.8%)	0.870
COPD, *n* (%)	5 (12.5%)	8 (10.0%)	0.567
Hypertension present, *n* (%)	9 (22.5%)	25 (31.3%)	0.317
Diabetes present, *n* (%)	10 (20.5%)	15 (18.8%)	0.478
Coronary heart disease present, *n* (%)	8 (20.0%)	22 (27.5%)	0.503
TNM classification, *n* (%)			0.610
I	16 (40.0%)	34 (42.5%)	
II	14 (35.0%)	25 (31.3%)	
III	10 (25.0%)	21 (26.3%)	
Intraoperative data			
Propofol consumption (µg)	821.62 ± 186.57	832.75 ± 142.83	0.236
Intraoperative fluid blood loss (ml)	62.31 ± 40.23	69.63 ± 43.95	0.413
Surgery time (min)	99.35 ± 39.27	97.46 ± 40.21	0.418

As given in Table 2, the cumulative consumption of intraoperative TCI remifentanil was significantly lower in the PVB group compared with the placebo group with a mean difference of 554.06 (95% CI: 145.88–962.52) (1,432.20 ± 383.53 vs. 878.14 ± 98.37, *p* = 0.014). In addition, the intraoperative IV fentanyl was also significantly different between the two groups (*p* < 0.001). The patients in the placebo group needed more fentanyl than those in the PVB group with a mean difference of 142.59 (95% CI: 95.52–189.68) (mean ± SD of 266.42 ± 41.97 and 123.83 ± 17.98, respectively).

**Table 2:** Comparison of intraoperative and postoperative opioid consumption, duration of first-time usage of PCIA, number of PCIA press, and rescue analgesia consumption between the two groups within 48 h following VATS LOBECTOMY.

Outcome	Control group (*n* = 40)	PVB group (*n* = 80)	Mean difference	95% CI	T/Z value	*p*
Intraoperative opioid consumption						
TCI remifentanil (ng, mean ± SD)	1,432.20 ± 383.53	878.14 ± 98.37	554.06	145.88–962.52	3.130	0.014
IV fentanyl (mcg, mean ± SD)	266.42 ± 41.97	123.83 ± 17.98	142.59	95.52–189.68	6.984	<0.001
Postoperative opioid consumption						
Sufentanil consumption (µg, mean ± SD)	289.64 ± 21.64	188.55 ± 19.97	101.09	95.22–106.95	33.985	<0.001
Duration of first-time usage of PCIA [min, median (IQR)]	40.19 ± 8.80	70.14 ± 10.92	29.96	27.16–32.75	21.142	<0.001
Number of PCIA press [median (IQR)]	4 (1–9)	2 (0–4)	—	—	581.0	0.034
Rescue analgetic consumption [mg, median (range)]	20 (0–80)	0 (0–60)	—	—	−2.743	0.006

In addition, the cumulative consumption of postoperative opioids in the PVB group was significantly lower compared with the placebo group with a mean difference of 101.09 (95% CI: 95.22–106.95) (mean ± SD of 188.55 ± 19.97 and 289.64 ± 21.64, *p* < 0.001). The median duration of first-time usage of PCIA was significantly longer in the PVB group compared with the placebo group with a mean difference of 29.96 (95% CI: 27.16–32.75) (median ± IQR of 70.14 ± 10.92 and 40.19 ± 8.80, *p* < 0.001). The number of PCIA press (times) for postoperative analgesia with 48 h was significantly decreased in the PVB group (*p* = 0.034). Consumption of rescue analgesia via intravascular parecoxib before removing of PCIA pump was lower in the PVB group compared with the placebo group [0 (0–60) vs. 20 (0–80), *p* = 0.006] (Table 2).

**Figure 3:**
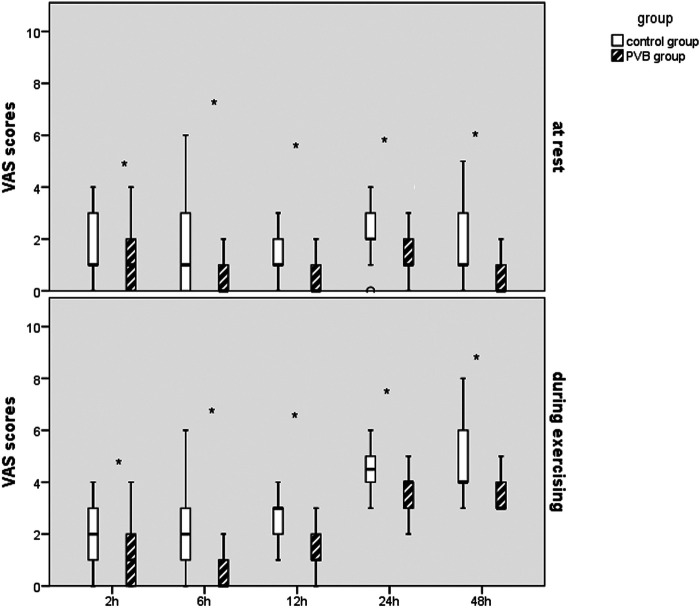
Results of the VAS scores in the two groups after VATS LOBECTOMY. *p < 0.05, compared with the placebo group.

Lower postoperative NRS scores at 2, 6, 12, 24, and 48 h at rest were observed in the PVB group compared with those in the placebo group, respectively. Significantly lower operative pain intensity assessed by NRS scores while exercising was, respectively, significant at 2, 6, 12, and 24 h after surgery in the PVB group compared with that in the placebo group, which was revealed by Mann–Whitney *U* test (Figure 3).

**Figure 4:**
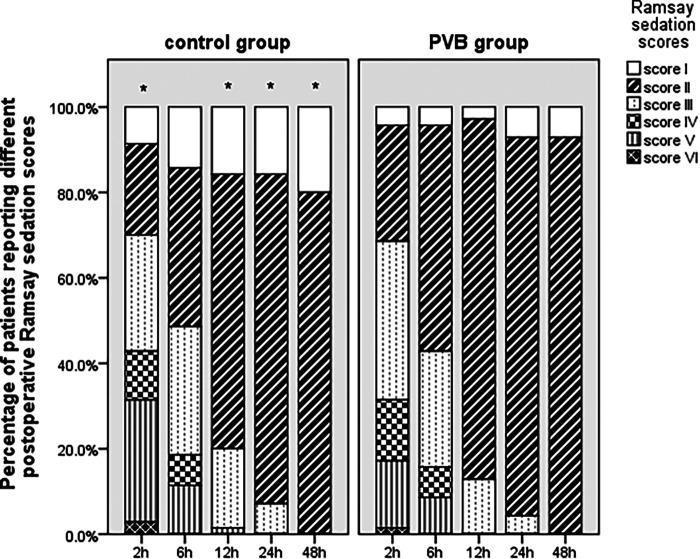
Postoperative Ramsay sedation scores at different intervals between two groups within the first 48 h following VATS LOBECTOMY. *p < 0.05 placebo group vs. PVB group.

Regarding the postoperative Ramsay sedation score as shown in Figure 4, proportion of patients who reported appropriate sedation was significantly higher in the PVB group than that in the placebo group at 2 (78.6% vs. 61.4%, *p* = 0.042), 12 (97.1% vs. 82.9%, *p* = 0.009), 24 (94.9% vs. 83.3%, *p* = 0.041), and 48 h (92.9% vs. 80.0%, *p* = 0.046) postoperatively, whereas no significant difference was observed at 6 h following VATS.

The total QoR-40 score was significantly increased in the PVB group compared with the placebo group, whereas the NRS pain score decreased in the two groups. In terms of data of different dimensions of QoR-40 scale, patients in the PVB group had a better total scale (*p* < 0.001), physical comfort scale (*p* < 0.001), and less pain (*p* < 0.001). However, the other dimensions such as psychological support, emotional state, and activity ability between the two groups did not reach the statistically significant difference (Table 3).

**Table 3:** Comparison of QoR-40 scores within 48 h after VATS LOBECTOMY between the two groups.

Group	Total	Physiology	Emotion	Cognition	Nociception	Activities of daily living
Control group (*n* = 40)	168.24 ± 20.03	44.66 ± 11.46	41.24 ± 20.03	39.33 ± 6.59	25.25 ± 9.64	20.23 ± 20.03
PVB group (*n* = 80)	180.67 ± 21.90*	59.25 ± 9.64*	43.55 ± 19.58	37.35 ± 10.33	34.66 ± 11.45*	22.51 ± 17.96

* *p* < 0.001 compared with the control group.

The difference in the occurrence of postoperative pulmonary complications after surgery was not significant between the two groups (2.5% vs. 3.8%, *p* = 1.000). PONV was the most common postoperative complication in the study, and it was more frequent in the placebo group (30.0% vs. 8.8%, *p* = 0.006). Higher incidence of postoperative hypotension occurred in the PVB group compared with the placebo group (7.5% vs. 5.0%), whereas no significant difference was observed between the two groups (*p* = 0.717). Although the patients in the PVB group have a decreasing trend in postoperative dizziness, pruritus, and delirium, no significant differences were observed between the two groups (Table 4).

**Table 4:** Comparison of the incidence of postoperative adverse events.

Variables	Control group (*n* = 40)	PVB group (*n* = 80)	*χ*^2^ value	*p*
Pulmonary dysfunction	1/40 (2.5%)	3/80 (3.8%)	0.129	1.000
Hypotension	2/40 (5.0%)	6/80 (7.5%)	0.268	0.717
Delirium	3/40 (7.5%)	2/80 (2.5%)	1.670	0.332
Dizziness	8/40 (20.0%)	6/80 (7.5%)	4.043	0.068
Pruritus	6/40 (15.0%)	5/80 (6.38%)	2.452	0.177
PONV	12/40 (30.0%)	7/80 (8.8%)	9.036	0.006

## Discussion

Although VATS has been regarded as the standard minimal invasive surgical procedure for lung resection since it was first introduced in 1992, a previous study has reported that moderate-to-severe acute pain remains a crucial problem in patients who underwent VATS due to utility incision, pulmonary parenchymal damage, muscular damage, pleural inflammation, and placement of chest tube, which can impair coughing, secretion clearing, forced vital capacity, and forced expiratory volume resulting in possible respiratory insufficiency, bronchial obstruction, and pulmonary infection (10, 11). In addition, approximately 34% of patients suffer from chronic pain attributed to VATS procedures. Therefore, it is critical to take aggressive MMA strategies to optimize intraoperative pain control to avoid the detrimental effects of opioid overuse and management of postoperative pain to reduce the likelihood of developing chronic pain and enhance recovery following VATS LOBECTOMY.

Currently, TEA remains the gold standard for analgesia with the highest degree of patients’ satisfaction following open thoracotomy (12). However, the performance of TEA requires a highly trained technique, and it can also result in both vasodilatation and cardiac depression causing hypotension due to bilateral sympathetic nerve block or urinary retention due to suppression of urination reflex, which limited its use. In addition, it is not suitable for patients with anticoagulant or blood clotting disorders, cardiovascular dysfunction, and previous spinal surgery (13). Recently, reviews have demonstrated that paravertebral block is a promising alternative to epidural block for its convenience and safety. It provides an equivalent effect in terms of controlling acute pain and reduces the risks of developing postoperative complications compared to TEA, specifically urinary retention and hypotension (14, 15). The triangular thoracic paravertebral space consists of the spinal nerves, intercostal nerves, and sympathetic nerve chains after leaving the intervertebral foramen, and injecting LA into this space can block the ipsilateral somatosensory and sympathetic nerves. Hence, respiratory and sympathetic function can be preserved on the contralateral side which is related to fewer pulmonary complications, less hypotension, and urinary retention compared with the thoracic epidural technique (16).

Vogt et al. conducted a double-blinded, prospective, randomized trial to test the hypothesis that a single-injection thoracic PVB at the sixth thoracic vertebra produced clinically significantly lower pain scores than PCIA alone up to 48 h after thoracoscopic surgery. They found that two dermatomes above and below the injected level were successfully blocked in most patients, which was consistent with the results of Cheema's research showing that pain sensation after thoracoscopic surgery could be sufficiently blocked by spread of 10–15 ml of LA into a mean sensory level of 2.2 segments above and 1.4 segments below the injected level (17, 18). In addition, the lateral spread of LA into the intercostal space after single-injection, cephalic–caudal spread covering many thoracic dermatomes will also develop, because the paravertebral space is contiguous with the intercostal and epidural space (19). A recent prospective randomized trial compared single-injection PVB, PVB catheter, or TEA for postoperative analgesia following VATS, single-injection PVB was faster and equally as effective as PVB catheter, and it led to similar patient satisfaction as TEA. Therefore, they recommended the single-injection PVB technique to be considered in patients not suitable candidates for TEA (20). We also advocate that in contrast to single-injection PVB which has less technique challenges, continuous PVB through a catheter will unnecessarily expose patients to additional risks to pleural disruption during PVB catheterization. Furthermore, the catheter and device which are specifically used for PVB are still conflicting up to now. Several previous studies reported that conventional or commercially available epidural catheter was indwelled in the paravertebral space as the paravertebral catheter leading to a 29.5% incidence of pleural disruption (21). Unfortunately, PVB catheter implantation was also contraindicated in patients as conduction of TEA. The failure rate was high for PVB catheter placement and management in clinical practice. This is because the effect of continuous analgesia of PVB has been proved to be associated with the appropriate location of the paravertebral catheter and the working diffusion of analgesic drugs among the paravertebral space which highly required established technology standard, well-trained practitioners, and well-equipped facility (22, 23). Rather than using continuous PVB, the previous study evaluated a multiple-space technique in which the spread of LA was considered sufficient for the somatic and sympathetic pain control from the trauma resulting from VATS LOBECTOMY. They found that multiple PVB contributed to a significantly lower intraoperative fentanyl consumption as compared with multiple subcutaneous saline injections (*p* < 0.01), and postoperative cumulative morphine consumption was also significantly less in the PVB group at all postoperative time points (*p* < 0.05 for 12 h and *p* < 0.01 for all other time points) (24). Consistent with the above-mentioned previous studies, our results strongly favored for an optimized intraoperative pain control with significantly reduced intraoperative consumption of TCI remifentanil and IV fentanyl by the application of two-space PBV injection at the beginning of the surgery. In addition, according to our results, the cumulative consumption of postoperative opioids through PCIA within the first postoperative 48 h was proved to be significantly lower in the PVB group compared with the placebo group. Cowie et al. compared the spread of contrast dye in single-injection PVB at the thoracic 6–7 segment with 20 ml contrast and multiple PVB at thoracic 3–4 and 7–8 segments with 10 ml of contrast in each, and the result revealed that contrast dye spread more extensively across the intercostal segments with 4.5 spaces (range, 2–10) covered with a single injection and six spaces (range, 2–8) covered with a dual-injection technique (*p* = 0.03). Therefore, multiple techniques at separate levels covered more thoracic dermatomes, which might explain the significant contribution to enhance the analgesic efficacy (25).

According to the previous literature, the effect of single-injection PVB with long-acting LA was expected to last 9 h to 38 h with an average of 23 h after VATS LOBECTOMY (26). A randomized controlled clinical trial performed a two-shot PVB with 20 ml of 0.375% ropivacaine at the thoracic interspace T4–T5 and T7–T8 combined with GA, and pain scores at rest at 4 and 24 h and on cough at 4 h were lower in the PVB/GA group compared with the GA group (*p* < 0.05). However, no difference in pain scores at rest at 48 h and on cough at 24 and 48 h was found (27). In our study, we excluded the potential clinical factor which influenced the efficacy of the nerve blocks in providing perioperative analgesia due to the comparable surgery durations between the two groups. Therefore, postoperative pain scores could reflect the efficacy of PVB analgesia. To prolong analgesia of PVB, the concentration of ropivacaine was increased to 0.5%. As we expected, the VAS score of the PVB group at rest and while exercising at all time points within the first 48 h after VATS LOBECTOMY was significantly lower than that of the placebo group, which illustrated the effect of multiple PVB using high concentration of ropivacaine persisted at least 48 h. Therefore, due to significantly effective pain relief, a significant reduction in postoperative PCIA sufentanil consumption was obtained after the application of upon completion of the VATS LOBECTOMY. In addition, the results of the first analgesia time through PCIA, the number of PCIA press (times), and postoperative rescue analgesia consumption were significantly better in the PVB group compared with that in the placebo group. Higher proportion of patients an reported appropriate sedation level in the PVB group than that in the placebo group at all time points following VATS because of receiving analgesia from multiple PVB at the end of the surgery. Patients in the PVB group also reported a better QoR-40 scores with a significantly better physical comfort scale and less pain than those in the placebo group. Although a higher incidence of postoperative hypotension was observed in the PVB group compared with that in the controls, a difference did not reach a statistical significance, whereas the incidence of POVN in the PVB group was significantly lower than that in the placebo group, which might be due to less consumption of sufentanil in the PVB group. All the above-mentioned results in the present study were consistent with the previous study which also supported the evidence that patients would also benefit from a reginal technique continued to the postoperative period, which could offer improved postoperative analgesia with less systemic opioid consumption to avoid the corresponding complications and rapid recovery from surgery (28).

As is well known, thoracic PVB under US guidance is a recent technique providing several advantages compared with conventional thoracic PVB utilizing surface landmarks such as enhanced reliability, and direct visualization of needle puncture in real-time image. However, the methods of identification of PVB by US guidance, some rare but serious complications associated with this technique such as pneumothorax, inadvertent vessel puncture, or injection are still the primary issues. In addition, a US-guided technique is an advance maneuver which highly depends on the experience of the operator (29). Therefore, it would be more challenging to perform repeated PVB at multiple thoracic nerve levels under US guidance. Considering the fully and magnifying exposure of the thoracic paravertebral structures under thoracoscopy after lung atrophy, it was easy to quickly complete multiple PVB via the intrathoracic approach under thoracoscopic direct vision by the surgeon before closing the chest. In the present study, the transmural pleura was chosen to advance the needle vertically at a depth of 0.5 cm to avoid the risk of nerve root damage and inadvertent spinal damage. To the best of our knowledge, the present study is the first to apply thoracoscopic-guided multi-point PVB at the beginning of the surgery with a combination of GA. Based on our results, we advocated that the performance of thoracic PVB under thoracoscopy guidance is a simpler, faster, and safer approach which might be integrated into MMA protocols for patients undergoing VATS LOBECTOMY to provide a successful opioid-sparing effect with optimized perioperative pain control and enhanced postoperative recovery. Moreover, this technique might be extended to other laparoscopy surgery if conducted at the lower thoracic paravertebral space because of the advantages of direct visualization under endoscopy guidance.

Some limitations in the present study were identified: First, VAS scores which were adopted to evaluate postoperative pain intensity were highly subjective. Second, although multiple PVBs under guidance needed less technique challenges, experience was still a dominant factor in such approach; therefore, more research were required with adjusted experience and learning curve in the future. Third, the results of the present study were from a single-center study and lack of comparison to other analgesic modalities; therefore, a well-designed multiple-center study to compare the PVB technique with other regional anesthesia should be performed in the future.

As an important component of MMA, the thoracoscopy-assisted positioning of the multiple thoracic PVB is simple and effective in controlling pain both intra- and postoperatively for VATS LOBECTOMY. It was also associated with the absence of detrimental effects due to opioid overuse and benefits of the early resumption of activity and physical function recovery. Therefore, it might represent a valuable analgesic strategy for VATS surgery compared with GA. However, well-designed RCTs should be performed in the future to assess the long-term postoperative outcomes based on knowledge gaps identified after completion of our study.

## Data Availability

The original contributions presented in the study are included in the article/Supplementary Material, further inquiries can be directed to the corresponding author.
